# 

**Published:** 1850-10

**Authors:** 


### CONTENTS

OF THE

### MEDICAL EXAMINER.

NEW SERIES—NO. LXX.—OCTOBER, 1850.

### ORIGINAL COMMUNICATIONS.

Contributions to Obstetrics, with Tabular Views, and Miscellaneous Practical Observations. By Henry A. Ramsay, M. D., Raysville, Georgia, - - - - - - 562

The Treatment of Acute Rheumatism, illustrated by cases taken from private practice. By Geo. L. Upshur, M. D., Norfolk, Va., Member of the American Medical Association, - - 574

Absorption of the Testes from Metastasis of Mumps. By Columbus Hixson, Student of Medicine, Middletown, Guernsey County, Ohio, - - - - - - - - - 582

Case of death from fractured Femur. Reported by S. B. Mills, M.D., Resident Physician, City Hospital, San Francisco, California, 583

### BIBLIOGRAPHICAL NOTICES.

Surgical Anatomy. By Joseph Maclise, Surgeon. With colored plates. Part III. To be completed in four parts. - - 584
Materia Medica and Therapeutics. By Thomas D. Mitchell, A. M., M. D., Professor of the Theory and Practice of Medicine in the Philadelphia College of Medicine, &c. &c. &c. - - - 589
The Diagnosis, Pathology, and Treatment of the Diseases of the Chest. By W. W. Gerhard, M. D., Lecturer on Clinical Medicine to the University of Pennsylvania, one of the Physicians to the Pennsylvania Hospital. Third edition, revised and enlarged, - - - - - - 595
Researches on the Natural History of Death. By Bennet Dowler, M. D., of New Orleans. - - - - 598
Human Physiology. By Robley Dunglison, M. D., Professor of the Institutes of Medicine in Jefferson Medical College, Philadelphia, Vice President of the Sydenham Society, London, &c. &c. With nearly five hundred illustrations. Seventh Edition. Thoroughly revised, and extensively modified and enlarged. In two volumes, - - - - - - 610
A Practical Handbook of Medical Chemistry. By John E. Bowman, Fellow of the Chemical Society, Demonstrator of Chemistry in King's College, London, and Author of "Practical Chemistry," 611
British and Foreign Medico-Chirurgical Review. July, 1850. American Edition, - - - - - - - 612

### EDITORIAL.

Commencement of the Winter Sessions, - - - - 613
Appointment of Drs. S. D. Gross and Elisha Bartlett, - - 613
Appointment of Dr. Baxley, of Baltimore, - - - - 613
Demonstrative Midwifery, again, - - - - - 614
Queries on the Influence of Tobacco, - - - - 614
Death of Dr. Hartshorne, - - - - - - 614
Military Surgeons in France.—Speech of Mr. Dupin, President of the National Assembly, - - - - - 615
Army order—relative rank of Army and Navy Officers, - - 616
Deaths in Philadelphia from Aug. 24th to Sept. 21st, 1850, - - 617

### RECORD OF MEDICAL SCIENCE.

### ANATOMY AND PHYSIOLOGY.

Comparative size and shape of the Thyroid Foramen in the Male and Female Innominatum, - - - - - - 618

### PATHOLOGY AND PRACTICE OF MEDICINE.

Case of Poisoning with Arsenic. Death in thirty-six hours—Influence of Arsenic on Digestion—Remission of Symptoms. By A. G. Greaves, Surgeon to the Derby Dispensary, - - - 619

Treatment of Singultus by Sulphuric Acid. By Dr. Schneider, - 621

On the Treatment of Hematemesis and Melæna. By Dr. J. M. Neligan, Physician to Jervis Street Hospital. Dublin, - - 621

On the Use of Ergotine in External and Internal Hemorrhages. By By M. J. Bonjean, Chambery, - - - - - 622

### SURGERY.

On "Ingrowing" of the Toe-Nail. By H. J. M'Dougal, Esq., - 623

### MATERIA MEDICA AND THERAPEUTICS.

Practical Remarks upon Ipecacuanha, with a formula for a more uniform and efficient preparation of the syrup, than that laid down in the Dispensatories. By Edward Jenner Cox, M. D., New Orleans, - - - - - - - - 623

### OBSTETRICS.

Turning by External Manipulation, - - - - . 625

